# Prenatal Origin of Pediatric Leukemia: Lessons From Hematopoietic Development

**DOI:** 10.3389/fcell.2020.618164

**Published:** 2021-01-12

**Authors:** Anna Cazzola, Giovanni Cazzaniga, Andrea Biondi, Raffaella Meneveri, Silvia Brunelli, Emanuele Azzoni

**Affiliations:** ^1^School of Medicine and Surgery, University of Milano-Bicocca, Milan, Italy; ^2^Centro Ricerca Tettamanti, University of Milano-Bicocca, Milan, Italy; ^3^Pediatrics, Fondazione MBBM/Ospedale San Gerardo, University of Milano-Bicocca, Milan, Italy

**Keywords:** pediatric leukemia, cell of origin, hematopoiesis, hematopoietic stem cells, eryhtro-myeloid progenitors

## Abstract

Several lines of evidence suggest that childhood leukemia, the most common cancer in young age, originates during *in utero* development. However, our knowledge of the cellular origin of this large and heterogeneous group of malignancies is still incomplete. The identification and characterization of their cell of origin is of crucial importance in order to define the processes that initiate and sustain disease progression, to refine faithful animal models and to identify novel therapeutic approaches. During embryogenesis, hematopoiesis takes place at different anatomical sites in sequential waves, and occurs in both a hematopoietic stem cell (HSC)-dependent and a HSC-independent fashion. Despite the recently described relevance and complexity of HSC-independent hematopoiesis, few studies have so far investigated its potential involvement in leukemogenesis. Here, we review the current knowledge on prenatal origin of leukemias in the context of recent insights in developmental hematopoiesis.

## Introduction

Leukemia is the most frequent childhood malignancy and it is characterized by a heterogeneous manifestation (Steliarova-Foucher et al., 2017). Despite improved clinical outcome in recent years, the incidence rate is increasing (Howlader et al., 2020). Therefore, there is an urgent need to develop precision medicine strategies for specific targeting of pediatric leukemias.

Even though the cellular origin of the childhood disease remains unclear, several lines of evidence suggest that its origin is distinct from that of the adult counterpart (Bolouri et al., 2018; Malard and Mohty, 2020). The hypothesis of a prenatal origin of pediatric leukemias was initially proposed in the mid-sixties (MacMahon and Levy, 1964) and various evidences are now supporting this view. Although hematopoietic stem cells (HSCs) sustain the production of most blood cells in adults, the ontogeny of vertebrate hematopoiesis is characterized by the presence of HSC-independent hematopoietic cells that originate sequentially during embryo development and in some cases persist to adulthood (Ghosn et al., 2019). These observations raise the hypothesis that HSC-independent progenitors arising at the embryonic level could be subject to genetic hits leading to childhood leukemia.

Herewith, we will focus on the established evidence of the *in utero* origin of the disease, and discuss about recent advances in the understanding of embryonic hematopoiesis, which is crucial for the identification of the still elusive origin and features of pre-leukemic clones. We will highlight the ontogeny impact on cell transformation focusing on both HSC-independent and HSC-dependent progenitors and report the recent insights into unique embryonic hematopoietic cell populations potentially providing a permissive environment for cell transformation.

## Childhood Acute Leukemias

Acute leukemias are characterized by uncontrolled proliferation of undifferentiated cells, called blasts, which impair normal hematopoiesis, in the bone marrow (BM) and peripheral blood (PB), with secondary infiltration of other tissues. According to morphology and cytochemistry, they are classified into acute myeloid leukemia (AML) and acute lymphoblastic leukemia (ALL). Less frequently, they can show an intermediate phenotype, with features of both diseases, and are defined as mixed lineage leukemias. During childhood, lymphoid phenotypes are predominant over myeloid ones, with a highest age-specific incidence between 2 and 5 years. On the contrary, the incidence of childhood AML is highest in patients younger than 1 year (infants) (Howlader et al., 2020). 5-year survival rate has increased over time and it currently reaches 90% and over 60% for childhood ALL and AML, respectively (Gatta et al., 2014; Malard and Mohty, 2020). Despite presenting with unique clinical and biological features, the outcome of infant AML patients is similar to that of older children (Masetti et al., 2015), whereas infants with ALL tend to manifest a more aggressive course of the disease with an event-free survival lower than 50% (Pieters et al., 2007).

Cytogenetic and molecular lesions diverge in infant, childhood, and adult acute leukemias in terms of type and recurrence. Chromosomal aberrations and especially translocations involving the gene *KMT2A* (*MLL*) are the most common genetic lesions in both AML and ALL infant leukemias, but are less frequent in children and adults (Malard and Mohty, 2020; Rice and Roy, 2020). Other frequent translocations in young children (<3 years) with AML involve *CBFA2T3* and *MNX1*, while *RUNX1*, *CBFB*, and *RARA* peak in older children (Bolouri et al., 2018). In infants with ALL, *ETV6*-*RUNX1* (*TEL*-*AML1*), and *TCF3-PBX1* rearrangements are prevalent, as well as a high-hyperdiploid karyotype (Hein et al., 2020; Malard and Mohty, 2020). Conversely, the *BCR-ABL* translocation is more frequent in adults with ALL (Malard and Mohty, 2020). Focal mutations in the *N/KRAS*, *KIT*, and *CBL* genes are more frequent in children than adults with AML, whereas *IDH1*, *IDH2*, *RUNX1*, *NPM1, DNMT3A*, and *TP53* mutations are almost exclusively found in the latter category (Papaemmanuil et al., 2016; Bolouri et al., 2018). On a similar line, the genomic landscapes of adult and childhood ALL differ (Liu et al., 2016; Gröbner et al., 2018).

Together, these observations led to speculate that childhood and adult leukemias are biologically distinct and might diverge not only in their molecular landscape but also in their cellular origin. Nevertheless, the underlying mechanisms for these differences and the precise entity of the cell(s) of origin of childhood acute leukemia are still unknown.

## Evidences for a Prenatal Origin of Childhood Leukemias

The hypothesis of a prenatal origin of childhood leukemias derived from studies on pairs of monozygotic monochorionic twins with both members affected by the disease, with the first case reported in 1882 (Greaves et al., 2003). A proposed explanation of leukemia concordance (shared disease features) in twins is that preleukemic cells arising in one twin fetus can diffuse *via* vascular anastomosis of a monochorionic placenta to the other twin (Clarkson and Boyse, 1971). The key piece of evidence which allowed to conclusively demonstrate prenatal initiation of leukemia was provided by the identification of unique clonal markers of leukemic cells, such as chromosome translocations, which can facilitate tracking of preleukemic clones. Interestingly, chromosome breakpoints always occur in a unique intronic region of the genes involved in the rearrangements and differ from patient to patient, although the fusion proteins generated are functionally equivalent. The evaluation of breakpoints in twins with concordant leukemia allowed to demonstrate that these children share the same breakpoints and consequently leukemia originated prenatally (Greaves, 1999). Preleukemic clones generated *in utero* have been described to evolve to overt leukemia either few days after birth or as long as 14 years later (Ford et al., 1993; Wiemels et al., 1999; Maia et al., 2004).

Rearrangements that involve the histone lysine methyltransferase 2A, *MLL*, at chromosome 11q23, are recurrent events in childhood leukemia, with highest incidence in infants (Wiemels et al., 2002). These rearrangements occur in approximately 50 and 70–80% of infant AML and ALL, respectively, and their frequency decreases with age (Hilden et al., 2006; Pieters et al., 2007; Harrison et al., 2010). Up to 135 *MLL* partner genes have been identified so far, among which *AFF1* (*AF4*), *MLLT3* (*AF9*), *MLLT10* (*AF10*), *MLLT1* (*ENL*), and *ELL* are the most prevalent (Meyer et al., 2018). Especially in infant ALL, and in approximately 60% of AML pediatric cases, *MLL* rearrangements seem sufficient to induce leukemic transformation on their own, since they usually not co-occur with additional mutations (Balgobind et al., 2011; Andersson et al., 2015). Retrospective analysis of blood spots taken at birth (Guthrie cards), which allow for the detection of around 1–20 leukemic cells in a single spot, have shown the acquisition of *MLL* translocations *in utero* (Gale et al., 1997), as already suggested by concordance studies on twins (Ford et al., 1993). Furthermore, the *in utero* appearance of cytogenetic lesions typical of leukemia has been supported by the detection of a *MLL*-fusion gene in fetal tissue and BM from abortions (Uckun et al., 1998). Another frequent gene fusion detected in pediatric leukemia involves the genes *ETV6*, on chromosome 12, and *RUNX1*, on chromosome 21. *ETV6*-*RUNX1* occurs in approximately 25% of B lineage pediatric ALL (Bernard et al., 1996). Monozygotic twins, which both developed ALL before their fifth birthday, have been described to share the same *ETV6*-*RUNX1* sequence (Ford et al., 1998). Additionally, after the evaluation of Guthrie cards of newly diagnosed ALL patients with *ETV6*-*RUNX1* fusion, both twins and singletons of 2–5 years, have been revealed to share unique or clonotypic sequence of the translocation (Wiemels et al., 1999). The *AML1-ETO* translocation (*RUNX1-RUNX1T1*) derives from the rearrangement of chromosomes 8 and 21 and has been shown to be the most common rearrangement in both children and adults with AML, suggesting its appearance as a postnatal event. Nevertheless, the prenatal occurrence of the translocation is supported by its detection in blood spots collected at birth, which were still available at the time of AML diagnosis (Wiemels et al., 2002). Altogether, the early onset of the disease, the high concordance rate between twin pairs (5–50% within the range of 0–15 years and less than 1% for adults) (MacMahon and Levy, 1964; Buekley et al., 1996; Greaves et al., 2003; Greaves, 2005), and the presence of the first mutation hit already at birth strongly suggest a prenatal origin of leukemia. More recent studies showed that bone marrow mesenchymal stem cells (MSC) derived from infants with acute leukemia harboring *ETV6*-*RUNX1*, *E2A-PBX1*, and *MLL*-rearrangements express the fusion genes, suggesting that rearrangements can also occur in early embryonic progenitors before hemogenic specification (Menendez et al., 2009; Shalapour et al., 2010). These and other evidences suggest that the first mutation event can take place at different levels along prenatal hematopoietic development.

## Insights Into Embryonic Hematopoiesis

A fine understanding of hematopoietic ontogeny is critical to determine the processes that initiate and sustain the progression of hematopoietic disorders. Increasing knowledge in the field of developmental hematopoiesis unraveled a complex organization of the hematopoietic system during gestation, and showed that embryonic/fetal and adult hematopoiesis differ in many aspects. In this regard, mouse, zebrafish, and chicken models have provided essential information on the dynamics of emergence of hematopoietic stem and progenitor cells in vertebrates (Dzierzak and Bigas, 2018). In adults, HSCs are located in the bone marrow niche and reside at the top of the hematopoietic hierarchy. In embryos, instead, several tissues harbor hematopoietic activity and HSCs appearance is preceded by the emergence of other progenitors endowed with various potency.

**FIGURE 1:**
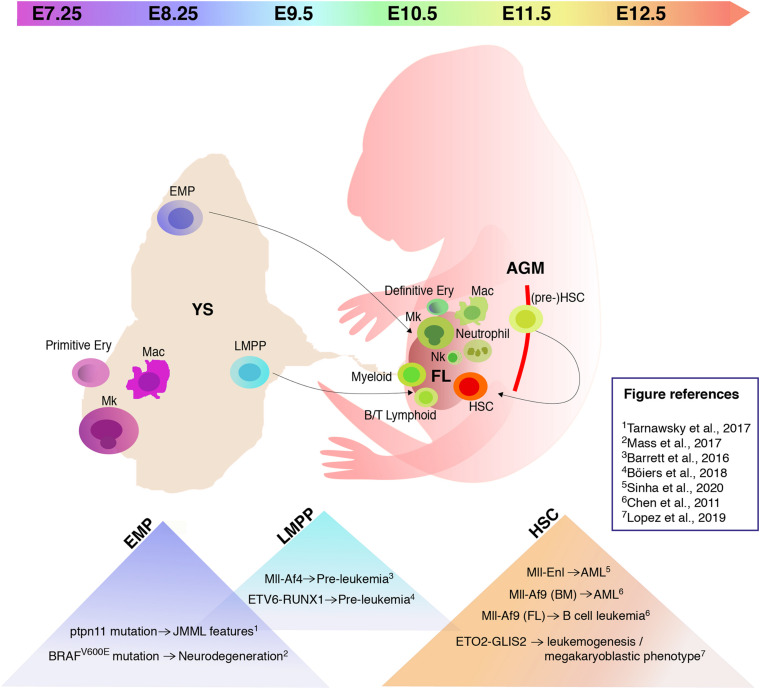
Hematopoiesis in the mouse embryo and embryonic hematopoietic cell populations that provide a permissive environment for cell transformation. Schematic of a mouse embryo showing sites of hematopoiesis (yolk sac/YS), fetal liver/FL, aorta-gonad-mesonephros/AGM) and timeline of appearance of hematopoietic stem/progenitor cells. The triangles in the lower part of the image list some recently studied mutations and translocations, and their effect following expression in specific embryonic hematopoietic cell populations. Mk, megakaryocytes; Mac, macrophages; EMP, erythromyeloid progenitors; LMPP, lymphoid primed multipotent progenitors; Ery, erythrocytes; Nk, natural killer; HSC, hematopoietic stem cells.

In vertebrates, embryonic hematopoietic development occurs in sequential waves (Figure 1). In the mouse, at embryonic day (E) 7.25 the extra-embryonic yolk sac (YS) represents the site of origin of the first hematopoietic wave which gives rise to primitive nucleated erythroid cells, macrophages (which do not transit through a monocyte intermediate), and megakaryocytes (Palis et al., 1999, 2001; Tober et al., 2007). A second wave originates from E8.25 in the YS and possibly other sites. Several progenitors with various potential are generated in this wave, including erythromyeloid progenitors (EMPs), lymphoid primed multipotent progenitors (LMPPs), and progenitors with multi-lineage mesodermal potential (Azzoni et al., 2014; Palis, 2016; Ghosn et al., 2019). All of these cells arise from hemogenic endothelium through a endothelial-to-hematopoietic transition (EHT) (Swiers et al., 2016; Ottersbach, 2019). EMPs differ in their surface marker profile from primitive hematopoietic cells and early definitive progenitors with HSC potential (Table 1). They possess myeloid potential and sustain erythropoiesis, megakaryocytes, and myeloid cell production, including macrophages and neutrophils (McGrath et al., 2015a). As more recently shown, they also have some lymphoid potential as they give rise to cytotoxic natural killer cells (NK) (Dege et al., 2020). Between E10.5 and E11.5, EMPs seed the fetal liver (FL) (Gomez Perdiguero et al., 2015; McGrath et al., 2015a). Similar to HSCs, EMPs are regulated by the c-Kit signaling pathway (Azzoni et al., 2018). Although EMPs were initially considered transient, it is now well accepted that they constitute a source of tissue-resident macrophages which persist and self-renew throughout adulthood, independently of HSCs (Schulz et al., 2012; Epelman et al., 2014; Gomez Perdiguero et al., 2015; Mass et al., 2016). Later than EMPs, but still before HSCs and FL hematopoiesis, LMPPs arise in the YS at around E9.5 (Figure 1 and Table 1). In the developing embryo they contribute to lymphopoiesis and myelopoiesis, and are devoid of the potential to generate erythrocytes, basophils, eosinophils, and tissue resident macrophages (Böiers et al., 2013). The HSC-independent wave is required for embryo survival (Chen M. J. et al., 2011). Given this relevance, it is possible that HSC-independent progenitors are subject to genetic hits leading to childhood leukemia.

**TABLE 1:** List of the main hematopoietic stem and progenitor cell populations in the early mouse embryo, their time and site of first emergence and surface marker expression profile.

**Cell type**	**Wave**	**First emergence**	**Surface marker expression**	**References**
Primitive erythroid cells	HSC-independent	E7.25 YS	TER119^*low/+*^ Kit^*low*^ CD41^*low*^	Palis et al., 1999; Ferkowicz et al., 2003
Primitive megakaryocytes	HSC-independent	E7.25 YS	CD41+GP1bβ+	Xu et al., 2001; Tober et al., 2007
Primitive macrophages	HSC-independent	E7.25 YS	CD45^+^ F4/80^+^ CD16/32^+^	McGrath et al., 2015a
EMPs	HSC-independent	E8.25 YS	CD41^+^ Kit^+^ CD16/32^+^ CD31^+^ Sca1^–^ Flt3^–^	McGrath et al., 2015a; Frame et al., 2016
LMPPs	HSC-independent	E9.5 YS	Lin^–^ CD45^+^ Kit^+^ Rag1^+^ Flt3^+^ IL7Rα^+^	Böiers et al., 2013
Pro-HSCs	HSC-dependent	E9.5 caudal part	Lin^–^ VE-Cadherin^+^ CD45^–^ CD43^–^ CD41^*low*^	Rybtsov et al., 2014
Pre-HSC type I	HSC-dependent	E10.5 AGM	Lin^–^ VE-Cadherin^+^ CD45^–^ CD43^+^ CD41^+^ CD201^+^	Rybtsov et al., 2011, 2014; Zhou et al., 2016
Pre-HSC type II	HSC-dependent	E10.5 AGM	Lin^–^ VE-Cadherin^+^ CD45^+^ CD43^+^ CD201^+^	Taoudi et al., 2008; Rybtsov et al., 2011, 2014; Zhou et al., 2016
FL HSCs	HSC-dependent	E12.5 fetal liver	Lin^–^ Sca1^+^ Kit^+^ CD45^+^ CD48^–^ CD150^+^ CD11b^*low*^ Flt3^+^ CD201^+^	Kim et al., 2006; Benz et al., 2012; Zhou et al., 2016

HSCs can be detected starting from E10.5 (Figure 1 and Table 1) in the aorta-gonad-mesonephros (AGM) region, budding from the ventral endothelium of the dorsal aorta through EHT (Müller et al., 1994; Medvinsky and Dzierzak, 1996; de Bruijn et al., 2000, 2002; Bertrand et al., 2010; Boisset et al., 2010; Kissa and Herbomel, 2010). Vitelline/umbilical arteries, embryonic head, and placenta represent additional embryonic vascular sites in which HSC activity takes place (de Bruijn et al., 2000; Ottersbach and Dzierzak, 2005; Robin et al., 2010; Ivanovs et al., 2011; Li et al., 2012). Until E12.5, very few transplantable HSCs arise in the embryo, although pro/pre-HSCs are already present from E9.5 (Rybtsov et al., 2016; Table 1). HSC maturation and expansion takes place in the FL from E12; subsequently, HSCs colonize the bone marrow where they reside throughout adult life (Kumaravelu et al., 2002; Rybtsov et al., 2016). Although it is well acknowledged that mammalian hematopoiesis is highly conserved and shares many similarities with the mouse (Enzan, 1964; Tavian et al., 2001; Ivanovs et al., 2011; Julien et al., 2016; Bian et al., 2020), the mechanisms that describe human hematopoiesis are less clear and need to be further investigated (Easterbrook et al., 2019).

## Models for the Study of the Origin of Childhood Leukemias

A comprehensive understanding of the cellular origins of childhood leukemia is fundamental for the establishment of faithful animal models. However, despite intensive investigation of the intrinsic and extrinsic factors regulating HSPC biology and their relationship to leukemogenesis, this has not been achieved so far. Several studies showed that gene fusions recurrent in pediatric leukemias can lead to divergent outcomes in terms of disease aggressiveness, latency, phenotype, and transcriptional features, according to their time of appearance during ontogeny (Chen W. et al., 2011; Horton et al., 2013; Man et al., 2016; Chaudhury et al., 2018; Lopez et al., 2019; Sinha et al., 2020). Differences in lineage specification and disease latency have been clearly shown after the induction of *Mll-Af9* in FL and adult BM HSCs. In these two models the translocation gave rise to B cell leukemia with a prolonged latency and AML, respectively (Chen W. et al., 2011). Similarly, transduction of *MLL-AF9* and *MLL-AF4* in human neonatal cord blood (CB) HSPCs mainly resulted in ALL (Horton et al., 2013; Lin et al., 2016). In contrast, *MLL-AF9* transduced human adult BM HSPCs gave rise to non-leukemic myeloid-biased engraftment and *MLL-Af4* transduced mouse adult BM cells led to AML (Horton et al., 2013; Lin et al., 2016). Consistent with these findings it has been demonstrated that B-cell committed progenitors harbor transforming potential in ALL, (Castor et al., 2005; Kong et al., 2008), but there are also evidences hinting at a cell of origin at an earlier developmental stage (le Viseur et al., 2008). The inducible expression of the recurrent gene fusion product *ETO2-GLIS2*, associated with acute megakaryoblastic leukemia, triggered leukemogenesis in both FL HSPCs and adult BM HSPCs, but gave rise to a megakaryoblastic phenotype with a shorter latency and caused more evident transcriptional changes only in the former (Lopez et al., 2019). The induction of the *Mll-Enl* translocation in FL HSPCs at E12.5 led to an overt, more aggressive AML form than the one triggered in adults, and to a transplantable disease in secondary recipients (Sinha et al., 2020). Overall, these studies suggested that childhood leukemias originating from the FL possess unique features that differentiate them from the ones resulting from the same genetic lesions occurring in the adult. However, they did not precisely address the cellular origin of the disease, as the FL is not a site of *de novo* hematopoietic generation. Cells seeding the FL during embryogenesis can have multiple origins, most of which are HSC-independent, and could already carry genetic lesions at the time of FL seeding. Besides the cell subtype in which the genetic hit takes place, the developmental stage of the hematopoietic niche also elicits an important contribution in regulating leukemic lineage commitment (Chaudhury et al., 2018; Rowe et al., 2019). Indeed, it has been recently shown how a *MLL*-rearrangement can differentially give rise to either a mixed lineage or a myeloid leukemia according to the developmental age of the microenvironment in a setting where the cell of origin is the same (Rowe et al., 2019). In addition to the fact that the susceptibility for transformation and the resulting phenotype change during ontogeny, differences in human and mouse embryonic hematopoietic development could also affect the faithful modeling of the human pediatric leukemia.

## An HSC-Independent Origin of Pediatric Leukemia?

The potential link between HSC-independent hematopoiesis and leukemogenesis remains so far largely unexplored. Only few studies have investigated which of the unique embryonic hematopoietic cell populations provide a permissive environment for cell transformation. As EMPs sustain fetal myelopoiesis and their progeny persists in the adult (Gomez Perdiguero et al., 2015; McGrath et al., 2015b), it has been recently suggested that EMPs may represent cells of origin of diseases associated with fetal development. Furthermore, in the context of the Mll-Af9 translocation, it has been shown how the proliferation rate is a limiting factor for malignant transformation (Chen et al., 2019). Thus, the rapid proliferation of EMPs (McGrath et al., 2015a) suggests that these cells may be susceptible to oncogenic transformation. A proof of principle of the notion that EMPs could be cells of origin for post-natal diseases derives from a recent study that investigated whether the neurodegeneration observed in patients with histiocytosis could be caused by somatic mutations in the EMP lineage. In particular, the mosaic expression of the BRAF^*V600E*^ mutation in EMPs at E8.5 was shown to cause expansion of microglia and neurodegeneration in adult mice (Mass et al., 2017). The involvement of HSC-independent hematopoietic progenitors has been assessed in the context of juvenile myelomonocytic leukemia (JMML). EMPs looked like good candidates, given the high relapse rates after HSCs transplantation (Locatelli et al., 2013) and the plausible *in utero* initiation of the disease (Matsuda et al., 2010). For this purpose, the ptpn11 gain-of-function JMML-initiating mutation had been introduced into EMPs. Although mice demonstrated features of JMML and mutant EMPs engrafted spleens of neonatal recipients, the disease was not transplantable (Tarnawsky et al., 2017).

Even though no studies have so far evaluated the susceptibility of EMPs to acute leukemias-specific genetic hits, some have specifically targeted acute leukemia-related translocations to LMPPs. LMPPs have recently emerged as potential cells of origin of B cell acute lymphoblastic leukemia (B-ALL) (Barrett et al., 2016; Böiers et al., 2018). Conditional activation of the *Mll-AF4* translocation in murine embryonic hematopoietic cells before the predominance of HSC-dependent hematopoiesis (E12.5–E14.5) resulted in a pre-leukemic phenotype. Even if mice showed B-cell lymphomas after a long latency, the model was unable to fully recapitulate the disease seen in patients harboring the same genetic alteration (Barrett et al., 2016). A possible counterpart for mouse LMPPs has also been identified in human FL as a IL-7R^+^ progenitor which maintains both myeloid and lymphoid potential. Evidence of the susceptibility of human fetal B cells progenitors to dysregulation by *ETV6*-*RUNX1* was provided by the introduction of the translocation in human induced pluripotent stem cells (iPSC). This led to a pre-leukemic initiation with expansion of the CD19^–^ IL-7R^+^ population, suggesting IL-7R progenitors as candidate cells of origin for *ETV6*-*RUNX1* preleukemia (Böiers et al., 2018). Moreover, the introduction of *Runx1* and *Ezh2* mutations in early thymic progenitors (ETPs), which are closely related to LMPPs, could model ETP leukemia features in mice and lead to acute lympho-myeloid leukemia progression upon introduction of *Flt3-ITD* (Booth et al., 2018). To summarize, recent work has shown that (i) HSC-independent progenitor cells can be subject to genetic hits occurring prenatally, which can lead at least to a pre-leukemic state; (ii) HSC-independent progenitors are susceptible to pre-leukemic initiation during a limited time frame, and (iii) multiple genetic hits can be required before an overt manifestation of the disease.

## Conclusion and Future Perspectives

Prenatal leukemic development in humans is a multifactorial process. Because of the complexity of the events leading to childhood acute leukemia and the difficulty of studying *in utero* stages, the identification and characterization of the cell(s) of origin is still a challenge. Although recent studies shed light on the potential of HSC-independent hematopoietic progenitor cells to act as the cell of origin for pediatric leukemia, there is urgent need to further investigate this aspect and in particular how leukemia-associated genetic hits may impact early stages of disease development *in utero*. This knowledge would be critical to better understand the etiology and pathogenesis of the disease, which would enable the refinement of animal models, the identification of new therapeutic approaches and to define preventive measures.

## Author Contributions

AC and EA conceived the topic, reviewed the literature, made the figure, and wrote the manuscript. GC, SB, AB, and RM revised and edited the manuscript. All authors read and approved the final version of the manuscript.

## Conflict of Interest

The authors declare that the research was conducted in the absence of any commercial or financial relationships that could be construed as a potential conflict of interest.
